# Stability of the core domain of p53: insights from computer simulations

**DOI:** 10.1186/1471-2105-9-S1-S17

**Published:** 2008-02-13

**Authors:** Arumugam Madhumalar, Derek John Smith, Chandra Verma

**Affiliations:** 1 Bioinformatics Institute, A-STAR (Agency for Science, Technology & Research), 30 Biopolis Street, #07-01 Matrix, Singapore 138671

## Abstract

**Background:**

The tumour suppressor protein p53 protein has a core domain that binds DNA and is the site for most oncogenic mutations. This domain is quite unstable compared to its homologs p63 and p73. Two key residues in the core domain of p53 (Tyr236, Thr253), have been mutated in-silico, to their equivalent residues in p63 (Phe238 and Ile255) and p73 (Phe238 and Ile255), with subsequent increase in stability of p53. Computational studies have been performed to examine the basis of instability in p53.

**Results:**

Molecular dynamics simulations suggest that mutations in p53 lead to increased conformational sampling of the phase space which stabilizes the system entropically. In contrast, reverse mutations, where p63 and p73 were mutated by replacing the Phe238 and Ile255 by Tyr and Thr respectively (as in p53), showed reduced conformational sampling although the change for p63 was much smaller than that for p73. Barriers to the rotation of sidechains containing aromatic rings at the core of the proteins were reduced several-fold when p53 was mutated; in contrast they increased when p73 was mutated and decreased by a small amount in p63. The rate of ring flipping of a Tyrosine residue at the boundary of two domains can be correlated with the change in stability, with implications for possible pathways of entry of agents that induce unfolding.

**Conclusion:**

A double mutation at the core of the DNA binding domain of p53 leads to enhanced stability by increasing the softness of the protein. A change from a highly directional polar interaction of the core residues Tyr236 and Thr253 to a non-directional apolar interaction between Phe and Ile respectively may enable the system to adapt more easily and thus increase its robustness to structural perturbations, giving it increased stability. This leads to enhanced conformational sampling which in turn is associated with an increased "softness" of the protein core. However the system seems to become more rigid at the periphery. The success of this methodology in reproducing the experimental trends in the stability of p53 suggests that it has the potential to complement structural studies for rapidly estimating changes in stability upon mutations and could be an additional tool in the design of specific classes of proteins.

## Background

p53 is a tumour suppressor protein that regulates the cell cycle and maintains the genomic integrity of the cell by orchestrating the activity of a wide variety of genes involved in repair, apoptosis and senescence [1-3] It is a multidomain protein and functions as a tetramer. Two homologous genes which are shown to share structural and functional homology with p53 are p63 and p73, whose isoforms are known to regulate some of the same apoptotic pathways that are also regulated by p53 [4-6]. These three proteins posses a modular architecture, constituted by an N-terminal transactivation domain, a DNA binding domain (DBD) and a regulatory C-terminal oligomerization domain [7,8]. The vast majority of tumour-derived p53 mutations map to the DBD [9]. The DBD mutations fall into two categories: (a) mutations that are at the DNA binding region of p53 and hence disable the binding of p53 to DNA and (b) mutations that alter the structural integrity and stability of p53 itself. The latter can cause local and global structural perturbations leading to the unfolding of p53 and so any process that can reverse this is likely to be of therapeutic value. It is known that the destabilizing effects of the latter can be countered by other mutations, the so-called second site suppressor mutations, and also by small molecules [10-19]. This underscores the importance of understanding the basis of the stability of this region.

**Figure 1:**
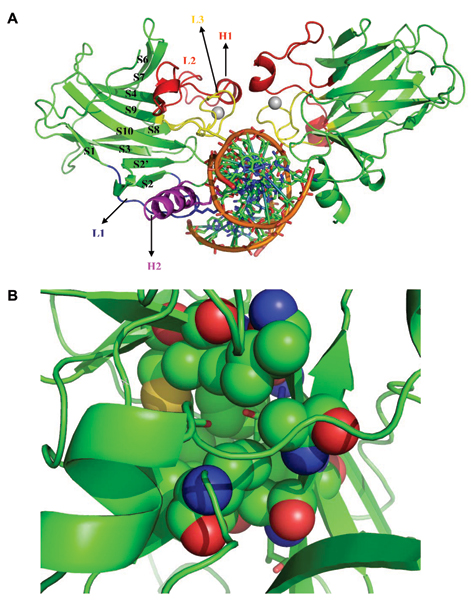
**Structure of the Core domain**. A. The structure of the p53 core domain in dimeric form complexed with DNA, taken from the crystal structure with RCSB code 2AHI [21]). Highlighted in magenta is the DNA binding helix H2, in red is the dimeric interface, blue is loop L1, yellow is loop L3. Zinc ion is represented as grey spheres; B. The two core residues Tyr236 and Thr253 shown in stick, surrounded in spheres by the predominantly hydrophobic core residues.

The DBD has been characterized structurally in complex with its cognate DNA by x-ray crystallography [20-22] and in its free form in solution by NMR [23]. The stability of isolated DBD has been found to be similar to that of intact p53 [24]. In addition, several crystal structures of the oncogenic mutants have also been solved to understand the structural basis for the inactivation of this domain [25-27]. The p53 core domain consists of a central β-sandwich that serves as a basic scaffold to which anchor the DNA binding surface and the surface of the dimeric partner DBD. Contacts to DNA are mediated through two large loops L2 and L3 held together by a Zn^2+ ^ion and a loop-sheet-helix motif (Fig. 1A). Contacts to the dimeric partner DBD are mediated through the H1 helix (shown in red Fig. 1). Zn^2+ ^ion is known to play a critical role in maintaining the structural stability and DNA binding specificity [28-32]. In spite of this seemingly stable architecture (highly packed β sheets), the core domain is known to be highly unstable, with a melting temperature of ~42–44°C [23]; the reasons for this are not yet understood. In contrast, the homologs p63 and p73 melt at higher temperatures (~61°C for p63; [33]). The core domain of p53 binds to different DNA sequences, depending on whether it enables the expression of genes that regulate cell cycle arrest or those that regulate apoptosis or indeed those that lead to the expression of other regulators. This process is further modulated by a host of other proteins that interact with the core domain too. Progress is now being made in understanding how this modulation occurs; for example there are proteins whose binding sites on the p53 core domain overlap with the binding sites of DNA sequences [34-37] There is some thought that the relatively low stability of the core domain is perhaps necessary to (a) enable rapid destruction of p53 in normal cells and (b) the rapid interactions with a multitude of proteins and DNA sequences in stressed cells. This clearly suggests that any changes in the core (or indeed other parts of the core domain) that will affect the stability of the core will translate into differing affinities for partner proteins and for the various DNA sequences and, hence the functions of p53 are bound to be affected [38]; however, a direct correlation remains to be established.

In an effort to understand the origins of instability of p53, Fersht & co-workers [23] noticed that the core is characterized by two polar residues, Tyr236 (located in strand S8) and Thr253 (located in strand S9) whose equivalents in p63/p73 are two apolar residues, Phe238 and Ile255. These two residues are two polar residues that are buried in an otherwise hydrophobic core of p53 DBD (Fig 1B). Hypothesizing that the presence of buried polar groups may incur a penalty that might destabilize p53, they replaced the two residues with the apolar equivalents from p63/p73 and found that the stability of mutant p53 had indeed increased (by ~1.6 kcal/mol). Analogous mutations that transform the core of p63 or p73 into that of p53 have not been reported in the literature.

In order to examine this problem computationally, we performed a set of studies that included building homology models of p63 and p73 (as there are no structures of these available in the public domain), and carried out molecular dynamics simulations and reaction path calculations to explore the basis of stability in p53, p63 and p73 and their mutants. We created double mutants of p53, replacing the Tyr236 and Thr253 by Phe and Ile respectively (here after referred as dp53) in the manner of Fersht & co-workers. In addition, we also mutated p63 and p73 by replacing their core residues Phe238 and Ile255 by the corresponding polar residues in p53 ie, Tyr and Thr (here after referred as dp63, dp73).

## Results

**Figure 2:**
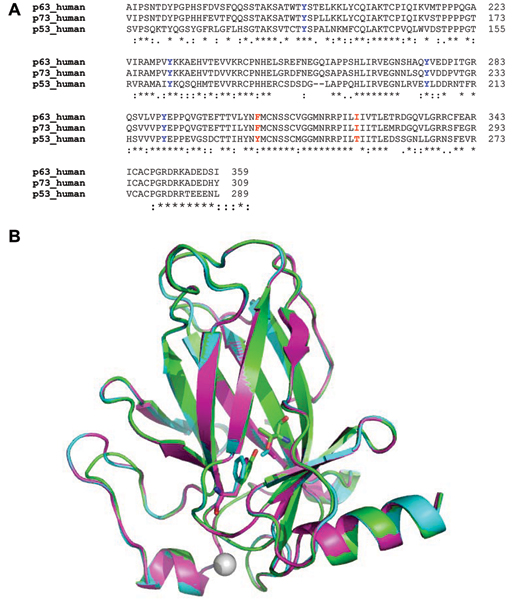
**Sequence alignment**. A. Multiple sequence alignment of the residues of the core domains of p53, p63, p73. Highlighted in red are the residues that have been mutated in this study and in blue are the Tyr residues whose mobilities have been examined. B. Superposition of the structures of the core domains of p53, p63 and p73 used in this study. Drawn in sticks are the residues that have been mutated in this study: Tyr236, Thr253.

The structural models of p63 and p73, as expected from the similarity of their sequences to p53, are very similar overall to the template structure of p53 (Fig. 2A, 2B). The Root Mean Squared Deviation (RMSD) over equivalent C-α atoms (194 in total) is 0.3 Å between p63/p73 and p53 and 0.2 Å between p63 and p73.

**Figure 3:**
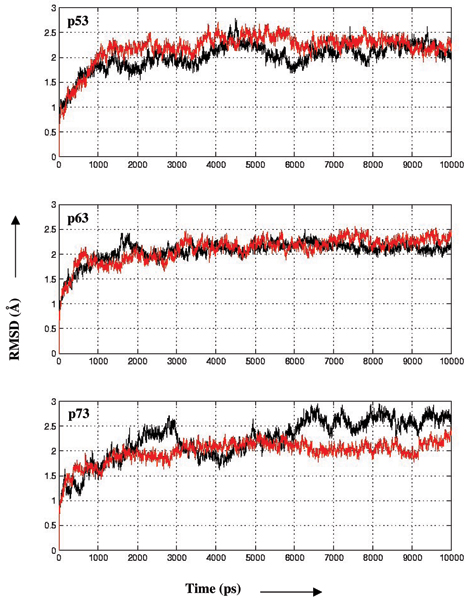
**RMSD**. Root Mean square Deviations (RMSD) of the backbone atoms as a function of time with respect to the starting structures during the MD simulations are shown for the wild type (black) and mutants (red) of p53, p63 and p73.

**Figure 4:**
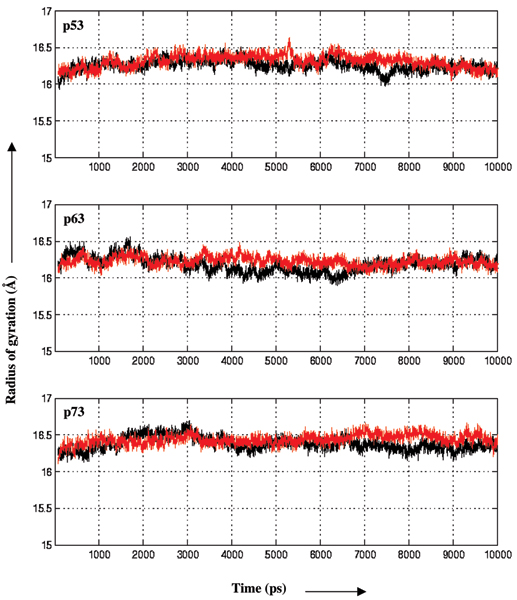
**Radius of gyration**. Radius of gyration as a function of time with respect to the starting structures during the MD simulations are shown for the wild type (black) and mutants (red) of p53, p63 and p73.

During the dynamics, the structural variations as measured by the RMSD and the radius of gyrations as a function of time for the 6 systems are shown in Fig. 3 and 4 and suggest that the simulations are stable. The mean RMSD values are 2.0(± 0.3), 2.2(± 0.3), 2.1(± 0.2), 2.0(± 0.3), 2.2(± 0.4) and 2.0(± 0.2) Å respectively for p53, dp53, p63, dp63, p73 and dp73. While the RMSD during the simulation has been used as an indicator of deviations from stability in some systems with an increase in the magnitude generally representative of destabilizing influences [18], we find no distinct pattern that indicates this in our systems. The only notable difference is seen in the wild type p73 simulation, which is due to the distortions of helix 2 of the DNA binding motif. The mean radius of gyration is 16.3(± 0.01), 16.3(± 0.01), 16.2(± 0.01), 16.2(± 0.01), 16.4(± 0.01 and 16.4(± 0.01) Å respectively for p53, dp53, p63, dp63, p73 and dp73. These results suggest that the simulations are stable and indicate that the globularity of the protein is retained throughout, without being affected significantly by the mutations. The similarity of the magnitudes of RMSD and radius of gyration of p53, p63, p73 shows that the similarity of the structures is reflected in global dynamics.

**Figure 5:**
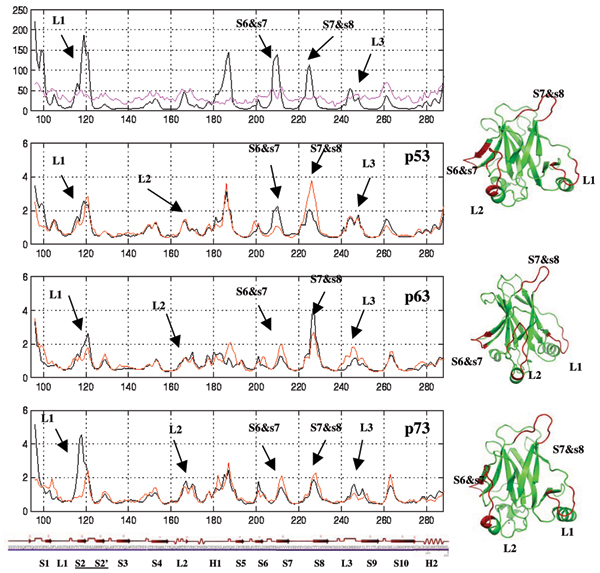
**Fluctuations**. Root mean squared fluctuations (RMSF) of the C-α atoms during the simulations shown for the wild type (black) and mutant (red) p53, p63 and p73. In the top panel the experimental B-factors (magenta) are shown in comparison with the RMSF of the p53 simulations.

The positional fluctuations of the C-α atoms (Fig. 5) show that the pattern of fluctuations in the wild type (and the mutant) p53 are similar to the experimental Bvalues (top panel of Fig 5). In all 3 systems, differences between the wild type and mutants are seen in the fluctuations in L1, L2, and the loops connecting S6–S7, S7–S8 and S9–S10. Patterns in L1, S6–S7 loop, and S9–S10 loop are similar between p63 and p73 while in L2, S7–S8 loop, L3 the patterns are similar between p53 and p73 (shown in the structures on the right in Fig 5). In the L1 region, between the wild type and mutants, while p63 and p73 show fluctuations that are larger than in the corresponding mutants, the fluctuations in L1 in dp53 are a little larger than in p53; the S6–S7 loop in p53 fluctuates almost twice as much as it does in dp53. In contrast the fluctuations in dp63 and dp73 are larger than in p63 and p73. The S7–S8 region in the mutants fluctuates more than in the wild type in p53 and p73 while it fluctuates less in p63. In L3 the pattern of fluctuations is similar in p53 wild type and mutant while they are larger in the mutant than in the wild type of p63 and in p73 they are larger in the wild type than in the mutant. The dynamics of loop L1(20–30) have been implicated in stability and in DNA binding from recent NMR studies [38] and L2 is involved in making contacts with the dimeric partner [20].

### Principal component analysis

**Figure 6:**
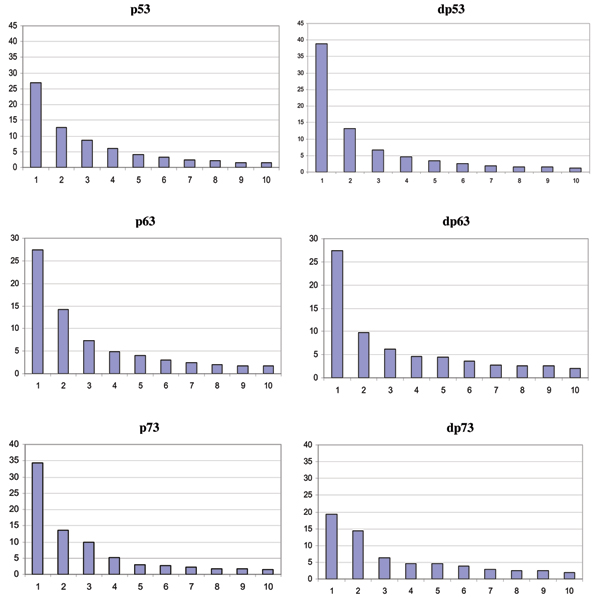
**Principal Component Analysis**. The percent contributions of the top 10 principal modes to the overall fluctuations during the simulations of wild type (left) and mutant (right) p53, p63 and p73.

**Figure 7:**
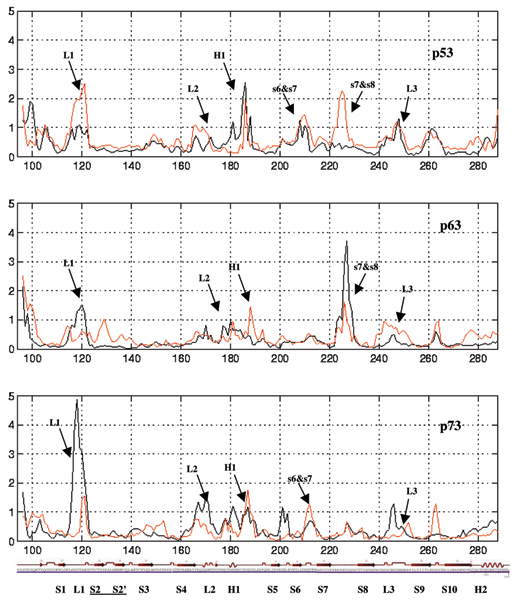
**Projections on PC1**. The RMSF of Cα atoms calculated after projecting the trajectories along their respective PC1 directions (black – wild type; red – mutant).

In order to further explore the nature of the fluctuations, principal component analyses (PCA) were carried out for all six systems. These yield a description of the dynamics of the protein in terms of the essential spaces (PCs) they inhabit [39]. It is clear that 80% of the fluctuations are captured by the top ten PCs (Fig. 6) and is generally a feature observed across a range of proteins [40]. PC1 contributes between 25–40% of the fluctuations with PC2 contributing about 12–15%. There is an increase in the contributions of PC1 in dp53 compared to that in p53. There is not much variation in PC1 in p63 (although there is ~40% reduction in PC2) while in p73 there is a decrease in PC1. This suggests at first glance that if the stability of p53 increases in the mutant form as has been demonstrated experimentally, then following the pattern of changes in the contributions of the PCs is indicative of a decrease in stability in both p63 (as judged by changes in PC2) and p73 (as judged by changes in PC1). When the trajectory is examined along PC1 (Fig 7) we see that motion in mutant p53 is mainly determined by large increases in fluctuations relative to those in the wild type. In contrast, in p63 and p73, the wild type proteins display larger fluctuations in general. The average RMSF of Cα atoms is 0.44 (± 0.4), 0.60 (± 0.5), 0.31 (± 0.5), 0.42 (± 0.4), 0.48 (± 0.6), 0.35 (± 0.3) Å for p53, dp53, p63, dp63, p73, dp73 respectively. The associated quasi-harmonic frequencies and entropies along PC1 and PC2 are shown in Table 1. These clearly show that the two frequencies in p53 increase upon mutation, leading thereby to an increase in entropy and thus stabilization of the free energy. In contrast the frequencies increase in p63 and p73 (in p63 they decrease marginally along PC2) thus leading to a decrease in the entropy and thus a destabilization of the free energy.

**Table 1:** The quasi-harmonic frequencies and associated entropies (cal/mol-K) of the top two principal components during the MD simulations

	**Frequencies cm**^-1^		**Entropy cal/mol-kelvin**	
	PC1	PC2	PC1	PC2

**p53**	0.95	1.57	12.69	11.69
**dp53**	0.94	1.50	12.70	11.78
**p63**	0.90	1.68	12.79	11.55
**dp63**	1.21	1.66	12.20	11.57
**p73**	0.79	1.20	13.06	12.21
**dp73**	0.9	1.39	12.80	11.93

**Figure 8:**
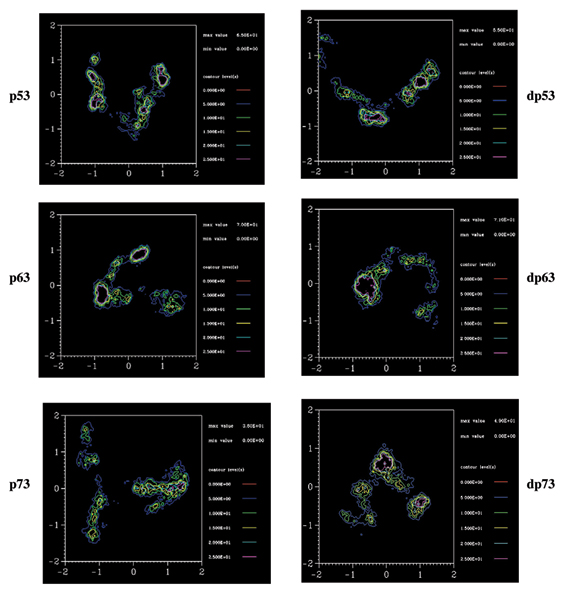
**Conformational Sampling**. The probability of sampling the phase space determined by principal modes 1 and 2 during the simulations of wild type (left) and mutant (right) p53, p63 and p73.

We further examine this through the probability of accessing regions of the phase space determined by PC1 and PC2 (Fig. 8). This shows two features: the more stable p63 and p73 do cover a larger region of phase space, particularly along PC1 compared to that covered by p53. In contrast, the mutants tend to show reduced coverage in p63/p73 and larger coverage in p53 compared to their wild types; in the case of p63 the differences are smaller than in p73. So far the data suggest that if the increases in flexibility in p53 correlate with the experimentally observed increase in stability upon mutations, then by analogy, we expect p73 to undergo a decrease in stability upon mutation; in the case of p63 it seems that the stability decrease will not be very pronounced.

### Structural plasticity

**Figure 9:**
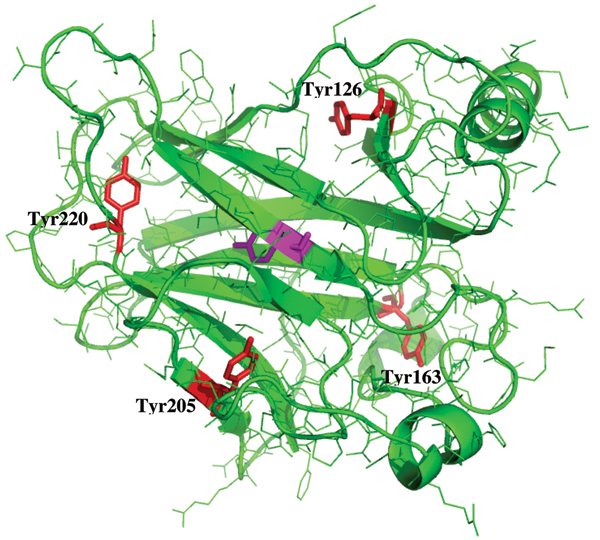
**Structural plasticity**. The structure of the core domain of p53 with the Tyr residues whose rates of flipping have been computed, shown as red sticks. Also shown is the core Tyr236 in magenta.

We next examine stability from a somewhat different perspective. This is through an examination of the nature of the experiments that are performed to study stability. Fersht's group studied stability by examining the accessibility of the proteins to increasing amounts of urea which would eventually lead to denaturation. This would require the urea molecules to penetrate to certain depths into the protein through pathways and induce unfolding [41]. This implies that the surfaces must be "pliable" or "penetrable" to different degrees. One method that is employed using NMR to explore such pathways is to explore the accessibility of residues to the solvent through exchange experiments while another technique, again using NMR, is to measure the timescales that characterize the rates of flipping of aromatic rings (Tyr and Phe). Indeed, Fersht and colleagues have examined such rates to characterize the nature of the core domain of p53 [23] and have reported a range of timescales that underlie a rich and complex dynamic behaviour. While the former method is relatively complex and beyond rigorous computational investigations, the latter can be examined using advanced techniques of simulations [42]. These motions are very good reporters of mobility but their timescales are far too high to be sampled in regular MD simulations and hence the need for more sophisticated methods. We apply one such technique, the Conjugate Peak Refinement method [43], that we have shown can effectively reproduce experimentally observed energetic barriers to such processes [44]. We have carried this out for the core Tyr/Phe residues that are the sites of mutations; in addition we have also computed these for 4 Tyr residues that are spread over the protein and are located towards the edges (Fig 9). This will enable some understanding of the nature of fluctuations that could possibly enable urea molecules to penetrate the surface of the protein. While a detailed analysis of our findings will be presented elsewhere, we summarize the key findings here.

The energies of the minimized structures are shown in Table 2. It is clear that these do not reflect the experimentally observed shifts in stability. We have augmented the enthalpies with a normal mode based vibrational entropy estimate and still observe the same lack of correlation with experimental stabilities. This is not surprising given that the initial structures are all derived from the one crystal structure of p53 (mutagenesis and homology based) and is constrained by the multiple minima problem; we have also attempted minimizations using a variety of other continuum models (Generalized Born in AMBER and CHARMM; data not shown) and they all show different shifts in energies and lack of correlations in trends compared to experimental data. This further highlights the importance of identifying alternative metrics that may give detailed insights into the origins of changes in stabilities.

**Table 2:** The enthalpies (with the noncovalent components) and the entropies of the minimum energy structures (in kcal/mol).

	**Enthalpy**	**Elec**	**Vdw**	**TΔS**
**p53**	-2421.9	-1660.9	-1494.5	-2061.5
**dp53**	-2417.8	-1660.4	-1490.9	-2061.6
**p63**	-2289.9	-1721.7	-1465.6	-2053.5
**dp63**	-2291.4	-1723.5	-1465.2	-2054.7
**p73**	-2254.6	-1671.2	-1465.2	-2065.9
**dp73**	-2256.9	-1672.5	-1464.9	-2066.6

**Figure 10:**
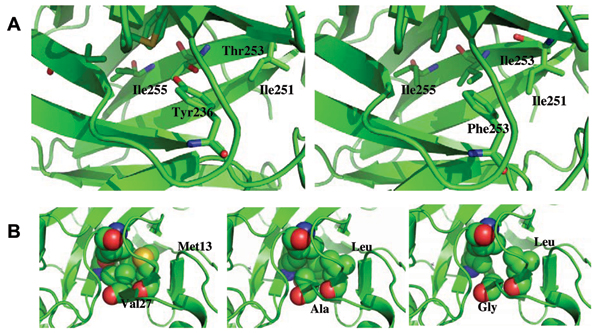
**The core region of wtp53 and dp53**. A. The residues in the core region of p53 (the left panel) and dp53 (the right panel) showing the differences in orientation of Ile251 and Ile255 when the Thr253 is mutated to Ile253 and Tyr236 is mutated to Phe236; B. The residues in the neighbourhood of the rotating ring that differ between p53 (Val, Met), p63 (Ala, Leu) and p73 (Gly, Leu).

The minimum energy pathways were computed for a set of Tyr residues located at the periphery of the molecule (Fig 9) and those at the core in all the 6 systems and the barrier heights are listed in Table 3. The barrier to rotation of the core Tyr236 of p53 is 11.1 kcal/mol and does not change much for the Tyr236Phe mutation (the barrier for the ring flip of Phe236 is 11.0 kcal/mol). However, when the Thr is mutated to Ile, the barrier for Tyr236 reduces to 4.0 kcal/mol and that for Phe236 in the double mutant Phe236-Ile253 reduces to 3.9 kcal/mol. This is linked largely to the unfavourable interactions between the polar sidechain of Thr253 and neighbouring Ile251 and Ile255 (Fig 10A). When the Thr is mutated to Ile, the cavity becomes apolar and the mobility that results enables the rotation of apolar Phe with less impedance and hence the reduced barrier height. This suggests that the presence of Thr236 leads to local rigidity at the core.

**Table 3:** Barriers to rotation of key Phe/Tyr residues (in kcal/mol)

	**Tyr236/Phe236**	**Tyr126**	**Tyr163**	**Tyr205**	**Tyr220**
**p53**	11.1 (11.0)	12.4	20.4	17.6	6.5
**dp53**	3.9 (4.0)	15.5	21.2	18.8	6.9
**p63**	3.9	13.6	16.4	6.8	7.7
**dp63**	3.0	17.4	9.5	8.5	7.0
**p73**	4.9	14.5	20.6	6.6	6.6
**dp73**	7.4	13.8	15.5	4.3	10.8

In contrast, the barriers for the equivalent Phe in p73 increases almost two-fold in the mutant while in p63 there is actually a decrease by 30%. What is interesting is that the barriers to rotation of the Phe in dp53 are smaller than the corresponding barriers in wtp63 and wtp73. Examining the local environment around the sites of rotation, we find that Val272 in p53 is Ala in p63 and Gly in p73 (Fig 10B), ie the cavity gets progressively less densely packed between p53, p63 and p73 and there is a correlated rise in the barrier height. The other difference in the immediate neighbourhood of the rotating ring is Met133 in p53 which is Leu in both p63 and p73. This suggests that packing helps to ease barriers to the complex processes of ring flips.

**Figure 11:**
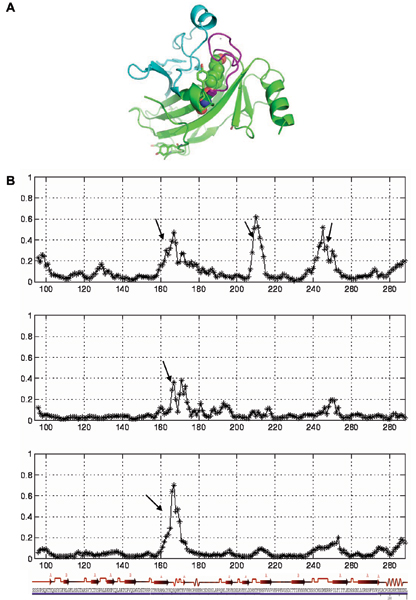
**Rotation of Tyr163**. A. The location of Tyr163 at the junction of two subdomains. The one consisting of L2 is in cyan and the subdomain containing L3 is in magenta. Tyr163 is shown in stick and Tyr236 and Thr253 are shown in spheres; B. The motion of the C-α atoms of the protein residues during the flip of Tyr163 for p53 (top panel), p63 (middle panel) and p73 (bottom panel).

The barriers that the four Tyr residues experience in p53 have increased in dp53. In contrast the barriers of two Tyrosines increase and those of the other two decrease in p63; in p73, the barriers of three of the four Tyrosines decrease. The variability in the barriers and the effects that the mutations have on them reflect the complex topography and underlying energy surface and reflects the differences in the packing in the proteins. However, Tyr163 lies at the junction of two sub-domains that could fluctuate, thus creating pathways for the entry of small solutes such as those that cause unfolding. One subdomain is formed by the region containing loop L2 and the other subdomain contains loop L3 (Fig 11A) One side of this region is constrained by a Zn atom while the other side is packed against each other through the Tyr163 sidechain. These are also the residues that move the most along the ring-flip reaction coordinate (Fig 11B). We know that in p53 the core becomes softer as judged by the decrease in barriers to ring rotation, and the periphery becomes more rigid as judged by the increase in barriers to ring rotation. This suggests that the mutant form would make it harder for the ligand urea to penetrate through to cause unfolding, leading thereby to the observed increase in stability. Again, if we extend this analogy to p73 where we saw that the pattern was similar to p53 (but in reverse) then, the core becomes more rigid upon mutation, while most of the periphery becomes softer and so upon mutations, it should be easier for urea molecules to penetrate the surface of p73 and cause destabilization than it would in the wild type. As seen in the PC analysis, it is again difficult to determine what happens in the case of p63. One thing is clear and that is that the core does become more rigid, but the periphery is more variable.

## Discussion

In an attempt to establish a structural and energetic basis for the low stability of the DNA binding domain of the tumour suppressor protein p53, we have carried out computational studies of the wild type p53 and its homologues p63 and p73 and their double mutants. The mutations have been guided by the sequence of the homologs p63 and p73 which are known to be more stable. Experimentally, the p53 mutant has been found to have increased stability although the biological activity is yet to be determined [23]. In an effort to correlate observations from simulations to experimentally observed stability issues in proteins, increasing deviation from the starting structures during the course of an MD simulation and, increased positional fluctuations have both been used as evidence for destabilizing influences on a protein's structure [18]). In our study however, we find that RMSD patterns do not correlate well with changing stabilities. Neither do the "free energies" of the structures that we have modelled. This could reflect both on the quality of our models and/or on the limitations of the force-fields [45]. Despite the overall similarity structurally and energetically (as judged by the fact that the sequences are highly similar and that the net charge is +3, +2 and +3 for p53, p63 and p73 respectively), we have seen that the systems are not equally "stable". A double mutant constructed experimentally [23], enhances the stability of p53. Our simulations suggest that this arises due to a net increase in fluctuations of the proteins. This would lead to an increased conformational sampling of the phase space which in turn leads to entropic stabilization of the overall free energy of the system. We see this increase when p53 is transformed into dp53, and we know that experimentally the stability of dp53 is increased [23]. We see a decrease when p73 is transformed into dp73 (the effect is not that pronounced for p63); there is no experimental data for this as yet. The pattern of changes seems to be largely determined by PC1 (and the associated entropic changes), which is the dominant mode of motion. Interestingly it is known that motions along the dominant mode are quite robust to sequence variations [46].

The residues that are under study here are located at the core of the protein suggesting that the increased stability in p53 (and decreased in p73) may arise from the removal (or introduction in p63 and p73) of buried polar groups. Several groups have been investigating the links between the nature of protein cores and overall stabilities. There are reports of increased rigidity associated with increased stability from MD simulations [18,47,48]. Lim and colleagues [49] report agreement between the MD simulations and the antibody-related observations on the nature of mutant structures; they also report agreement with the experimental observations of the change in DNA binding activity of some mutants. Our own observations suggest that enhanced sampling of phase space is linked to increased stability. This issue is as yet unresolved. The effects of core residue modifications upon protein stability remain unresolved. Some studies point out that burying polar groups increases the packing densities of proteins which in turn have a favourable effect on protein stability [50]. Other work has also concluded that burying polar groups leads to increased entropic stabilizations [51]. In contrast, there is other evidence that burying polar group can also destabilize proteins [23,52,53]. The observation by Lane & colleagues [54] that mutations of several surface residues of the DNA binding domain of p53 can have remarkable effects on its stability further highlights the complex nature of the stability issue. The picture is made more complex by observations that certain mutations at the cores of proteins lead to rearrangements that cause partial collapse to offset the size changes and minimize free energies, while in some cases, rearrangements expose polar groups that then attract solvent water from the bulk [55,56]. Matthews & colleagues [52] suggest that the landscape underlying such changes is characterized by a complex interaction between fluid like sidechain motions and more rigid peptide backbone motions. Clearly while there is some correlation between core rigidity, packing and overall stability, the issue seems to be far more complex and requires further detailed investigations [57].

Proteins are complex systems and while the nature of core residues will certainly dominate the overall rigidity, stability is a global property and there will be several other factors that contribute, as has been highlighted for the DNA binding domain of p53 by Lane & colleagues [54]. Our own simulations do point out certain features that seem to be consistent with experimental observations indirectly: the fluctuations of the helical segment that is part of Loop L2 (in both the C-α fluctuations of the original trajectory and in the trajectory along PC1, see Figures 6, 7) only for p53 and p73. The importance of Helix H1 in both p53 and p73 in DNA binding has been reported [58]. If we look at Figure 1, we notice that this region is the one that is involved in the protein-protein dimeric interface thereby hinting at the importance perhaps of dimerization and cooperativity in DNA binding [33].

In an attempt to understand the root of stability changes in a somewhat different manner, we examined in detail the experiments that have been used to probe stability. These experiments are related to the amount of urea needed to unfold a protein. This requires an understanding of the dynamics of parts of the protein which will form the pathways of entry of urea to the core of the protein [51]. While an exhaustive understanding of the various pathways is not available, we begin the process by examining the mobilities at various locations on the protein surface by computing the energetic barriers that characterize ring flips; such flips cause sufficient local deformation to enable openings for solvent molecules to enter the protein [[59]; see ]. While it is hard to estimate the rates of the processes associated with these ring flips as we have no entropic estimates of the transition states [except in certain cases – see for example [44]], it is clear from our studies here that (a) the rate at the core increases with increasing stability of the protein; (b) a range of time scales characterize the dynamics of the various parts of the protein; (c) the motions at the surface are very local and uncoupled from each other [23].

The residues that differ in the neighbourhood of the Tyr rings are: (a) for Tyr126: Pro128 in p53 and p73 is replaced by Thr128 in p63; Asn36 in p53 is replaced by Lys131 in p63 and p73; (b) for Tyr163: Glu171 in p53 and p63 is replaced by Asp171; (c) for Tyr205: Val203 in p53 is replaced by Ala205 in p63 and Val205 in p73; (d) there is a complex interplay of varying timescale motions across the protein surface; while the double mutant of p53 witnesses a dramatic reduction in the rate of flipping of the core aromatic sidechain, suggestive of increasing softness of the protein, the effects on residues that are towards the periphery (Tyr126, Tyr163, Tyr205 and Tyr225) in p53 are one of increasing the barriers to transitions – suggestive of increase in local packing or decreasing "softness". It is clear that small changes in the immediate environments of the rotating rings can affect the local packing in a manner that is reflected in the wide range of barrier heights. This does suggest that despite the fact that the ring flip itself is largely governed by local effects, somehow there are more global influences of the mutations that result in some "tighter" peripheral spots. This may form the basis of the need for larger amounts of urea needed to penetrate through the protein leading to the observed increase in stability. Additionally, two of the rings that we have studied, Tyr126 and Tyr163 are located in the vicinity of the DNA binding and the dimerization surfaces. Analyses of the ring flips and the associated movements clearly show motions that are likely to influence both these interactions (details to be presented elsewhere). It is clear that plasticity of the core residues is communicated to the dynamics of residues at the periphery. These will include those that mediate binding to DNA. How exactly this happens remains to be uncovered.

## Conclusion

In conclusion, we find that computational estimates of stability of proteins through their minimized energies partially reproduce experimental trends and may thus be a reasonable metric. Differences in root mean squared deviations over the course of MD simulations do provide some hints at changes in stability, as observed by Pan et al. [18]; however in our studies, this metric is not entirely discriminating. In our simulations, the enhanced sampling of phase space, dominated by motion along PC1, seems to be responsible for increasing stability. In addition, we have, for the first time to the best of our knowledge, applied methods of activated dynamics to understand protein stability as defined by urea induced unfolding. The mobility at the core of the protein is increased in systems of larger mobility as evidenced from higher rates of ring flips of aromatic residues; this suggests that larger conformational sampling increases the softness of the protein core, thereby making it more robust to structural perturbations. This seems to arise from a change of directed polar interactions to nondirectional apolar interactions. We find that the changes in mobility in surface regions of the protein and access to urea molecules correlates well with changing stabilities in p53 and perhaps in p73. While we do not yet have a measure of transforming these results into quantitative differences between experimental stabilities, we are applying this method to a range of other p53 mutants and other proteins to examine its validity and robustness. Initial results suggest that the method seems to hold the potential to rapidly estimate, at least qualitatively, the effects on the stability of proteins (at least in cases where there are ring-bearing residues at the periphery). If more generally valid, this method may well reduce the number of experiments that need to be carried out to examine the effects of mutagenesis on the stabilities, at least of a class of proteins, and would be an additional tool in protein design strategies.

A concluding point is about the two different force fields used in our analysis. We started the study using AMBER. However as pointed out, at the end of the MD study, we decided to expand the investigations by using methods of reaction paths (TRAVel) in order to explore the origins of stability as measured by urea-induced unfolding. These algorithms currently are only available in CHARMM. However the general differences amongst different force fields is quite small, as has been pointed out in a recent study [59] leading us to conclude that had we conducted our simulations using CHARMM, the overall conclusions would have been similar to those that we have reported using AMBER.

## Methods

The initial structure of monomeric p53 core domain was taken from the crystal structure of p53 bound to DNA (RCSB entry 1TUP resolved at 2.2 Å; [20]); the structures of p63 and p73 were modeled based on the homology with the p53 monomer (sequence similarity to p63 and p73 is 77% and 75% respectively while identity is ~60%). An alignment of the known structure against the sequences was generated by CLUSTALW [61], followed by manual manipulation using QUANTA [62]. The program MODELLER [63] was used to generate 20 initial homology models of the p63 and p73 DBD based upon the resulting sequence-structure alignment. The model with the lowest objective function was chosen as the representative model for further study. The double mutants of p53 (Phe236 and Ile253) (referred as dp53), p63 (Tyr238 and Thr255) (referred as dp63), p73 (Phe238 and Ile255) (referred as dp73) were made using QUANTA.

MD simulations were carried out using the AMBER [64] package. In all the four systems, the Zn ion was coordinated to three Cys residues and one His residue and the parameters for this pseudobond were taken from earlier studies [65,66]. Each system was solvated with TIP3P water box with the minimum distance of 10 Å to any protein atom. The positive charges in the system were balanced by adding chloride ions. The total number of atoms were 32617(p53), 32645(p63), 31491 (p73), 32621(dp53), 32644(dp63) and 31490(dp73).Parm99 force field was used for intermolecular interactions. Particle Mesh Ewald method (PME) [67] was used for treating the long range electrostatics. All bonds involving hydrogen were constrained by SHAKE. Time step of 2fs was used for dynamics integration. Before starting the dynamics, the whole system was minimized for 2000 steps, to remove any unfavourable interactions between the protein and solvent. The system was heated to 300K within 75 ps, under NPT conditions. Each system was simulated for 10ns at constant temperature (300K) and pressure (1 atm) [68] and the structures were stored every 1ps.

Reaction path calculations were carried out using the Conjugate Peak Refinement [43] algorithm as implemented in the module TRAVel in CHARMM [69]; this method is very robust and is currently only available in the program CHARMM. The protocol followed is the same as outlined before [44]. Briefly this consists of minimizing each system under a radius-dependant dielectric continuum model with an attenuation factor of 2 and with the non-bonded interactions shifted to zero between 8 and 12 Å. Minimizations were carried out until the change in gradient of potential energy was smaller than 10^-5 ^kcal mol^-1 ^Å ^-1^. Vibrational entropies were computed using the VIBRan module of CHARMM. This required the diagonalization of the full Hessian of the system [70]. The minimum energy state is then defined as the reactant state and the product state is created by simply interchanging the positions of the ring carbon atoms of the benzene ring of Phe/Tyr as the ring flip leads to a symmetric state. The minimum energy path between the reactant and the product state is then calculated using the TRAVel module which yields the saddle point(s) or the transition state(s), and the energy of the highest transition state is taken to tbe the rate limiting barrier height for that particular ring flip process.

## Competing interests

The authors declare that they have no competing interests.

## Authors' contributions

Madhumalar and Chandra conceived of the idea and wrote the manuscript; Derek carried out the homology modelings; Madhumalar carried out the MD studies; Chandra carried out the reaction path studies.
